# Spatio‐Temporal Changes in Effective Population Size in an Expanding Metapopulation of Eurasian Otters

**DOI:** 10.1111/eva.70067

**Published:** 2025-01-17

**Authors:** Nia Evelyn Thomas, Elizabeth A. Chadwick, Michael W. Bruford, Frank Hailer

**Affiliations:** ^1^ Organisms and Environment, School of Biosciences and Water Research Institute Cardiff University Wales UK; ^2^ Cardiff University ‐ Institute of Zoology Joint Laboratory for Biocomplexity Research (CIBR) Chinese Academy of Sciences Beijing China

**Keywords:** effective population size, Eurasian otter, genetic monitoring, *Lutra lutra*, population bottleneck, population recovery

## Abstract

Conservation efforts are leading to demographic growth and spatial expansion of some previously endangered species. However, past population bottlenecks or population size fluctuations can have lasting effects on effective population size (*N*_e_), even when census size (*N*_c_) appears large or recovered. The UK metapopulation of Eurasian otters ( *Lutra lutra* ) has a well‐documented history of population recovery over recent decades, with indicators of presence (faeces and footprints) increasing in distribution and number over successive national surveys. To determine whether this increase in *N*_c_ is reflected in increased *N*_e_, we analysed a large‐scale microsatellite dataset (21 years: 1993–2014; 407 individuals) for signals of recent *N*_e_ change using BOTTLENECK and LDNe, and evaluated potential biases associated with unaccounted spatial genetic structuring and inclusion of admixed genotypes. We obtained clear bottleneck signals in East England, and signals of recent population expansion in Wales and South West England in some analyses, consistent with national otter surveys and recent findings from whole‐genome sequencing. Analyses that did not account for spatial genetic structuring yielded strong spurious signals of United Kingdom‐wide population expansion, and *N*_e_ estimates from these analyses were suppressed by a factor of 3–4. Inclusion of admixed individuals had weaker impacts on *N*_e_ estimates, with overlapping 95% confidence intervals from different analyses. Notably, total *N*_e_ summed across regions was small and well below the *N*_e_ = 500 size deemed necessary for long‐term population viability (sum of river basin district groups: 170.6, 95% C.I.: 102.1–348.3). Conclusions drawn from UK otter surveys, which had suggested a robust population close to panmixia, are therefore not supported by our genetic evidence. Our study highlights the value of including genetic monitoring of endangered or recovering species in monitoring plans, while also providing methodologically important information about *N*_e_ estimation from real‐world datasets.

## 1 Introduction

Understanding and quantifying past and current population dynamics is one of the key goals of many conservation studies, but can be difficult to achieve, particularly for elusive species that are hard to monitor in the field. Genetic data can provide an important perspective for monitoring such species. Whereas census population size (*N*_c_) describes the total number of individuals in the population (or more narrowly the ‘number of adults alive at a given time’; Waples 2024), effective population size (*N*_e_) describes the number of individuals in an idealised population (e.g., Wright 1931) that would experience the same rate of change of allele frequencies as the census population (Charlesworth 2009; Wang, Santiago, and Caballero 2016), thus providing insights into the magnitude of inbreeding and genetic drift. Due to a range of factors such as temporal variation, or variation in breeding success among individuals and sexes, *N*_e_ tends to be smaller than *N*_c_ in wild populations (Ryman, Laikre, and Hössjer 2019; Hoban et al. 2021; Waples 2024).

The importance of *N*_e_ as a key parameter in measuring the maintenance of genetic diversity is exemplified by the call for its inclusion in the United Nations Convention of Biodiversity 2020 targets (Hoban et al. 2020), feeding into the Kunming‐Montreal Global Biodiversity Framework adopted by the Convention of Biodiversity in 2022 (Convention of Biodiversity 2022). *N*_e_ of a population or species is thought to be positively associated with reduced susceptibility to stochastic processes (Cristescu et al. 2010), with increased adaptive potential (Palstra and Ruzzante 2008) and therefore with increasing probability of survival (Frankham 1995a). Populations that have been through a bottleneck or a significant reduction in size are stochastically more susceptible to adverse extrinsic events and show reduced genetic diversity and *N*_e_. Population bottlenecks reduce both *N*_c_ and *N*_e_, and in the absence of immigration are predicted to decrease the genetic diversity of the population. Sequential bottlenecks or fluctuations in population size may lead to situations where—despite having a large contemporary overall size (*N*_c_)—a population remains at risk due to persistent low *N*_e_ (Frankham 1995a). Laboratory studies have shown that past bottlenecks can affect the extinction risk of a population even after it recovers to its previous size (Bijlsma, Bundgaard, and Boerema 2000), therefore estimating *N*_e_ and from this deducing which populations are at greater risk of extinction irrespective of their current *N*_c_ are important for wildlife managers and conservationists.

### 1.1 Population Size Estimation

For some species, *N*_c_ is relatively easy to determine via direct observation of a population, but until relatively recently *N*_e_ was much harder to calculate, as detailed data on breeding success are required for its estimation from demographic models (Leberg 2005). The rapid development of genetic approaches in recent decades means that *N*_e_ can nowadays be directly estimated from genetic data (Harris and Allendorf 1989; Luikart et al. 2010; Palstra and Fraser 2012; Hoban et al. 2021), with the caveat that different methods can result in different estimates due to varying assumptions, and confidence intervals can be large. For species that are elusive or live at low densities, such as otters, direct observation of *N*_c_ is problematic, and estimates of *N*_e_ using genetic data are now more achievable. Therefore, a ratio is often applied to translate estimates of *N*_e_ from genetic data into estimates of *N*_c_ (Frankham 1995b). Across studies, the modal estimate of *N*_c_ has been found to be larger than *N*_e_, typically by a factor of circa 10–11× (Frankham 1995b; Hoban et al. 2021), albeit with a wide variance (Waples et al. 2013; Clarke et al. 2024).

Estimating *N*_e_ from genetic data has mainly been achieved by using, where possible, two‐sample or temporal methods which used data taken at two points in time, preferably multiple generations apart, to detect changes in allele frequencies caused by genetic drift and thus produce an estimate for *N*_e_. These two‐sample estimators have employed a number of methods including temporal F‐statistics (*N*_e_ estimator, Do et al. 2014; TempoFs, Jorde and Ryman 2007), pseudo–maximum‐likelihood methods (MLNE, Wang 2001) and coalescent‐based Bayesian methods (TM3, Berthier et al. 2002 and CoNe, Anderson 2005). However, this requirement for two sets of genetic data, generations apart, can be problematic for species or populations that are not routinely monitored (and genetically sampled), or which have long generation times. Consequently, a set of methods that require data from only one time point, known as one‐sample estimators, have been developed. These estimators take a variety of different approaches to estimating *N*_e_, including approximate Bayesian computation (ABC) (ONeSAMP, Tallmon et al. 2008), sibship assignment or parentage (Colony2, Wang 2009 and AgeStruct, Wang et al. 2010) and linkage disequilibrium (LD) (LDNe, Waples and Do 2008). However, both one‐sample and two‐sample estimators of *N*_e_ assume discrete generations, which can be problematic in many sampling regimes and for species which show temporally overlapping generations (but see Waples, Antao, and Luikart 2014). Recent studies into the relative performance of various methods of *N*_e_ estimation showed that the LDNe approach provides a robust single‐sample estimator (Gilbert and Whitlock 2015), albeit sensitive to some factors such as mixture LD and Wahlund effects (see below).

### 1.2 Detecting Population Size Changes Using Genetic Data

Several methods have been developed to detect past population size changes using genetic data but can have differing efficacy depending on the timeframe of change. Both MSVAR (Beaumont 1999) (which uses likelihood‐based methods coupled with Monte Carlo integration) and ABCtoolbox (Wegmann et al. 2010) (which uses approximate Bayesian computation, ABC) are based on coalescent theory (Kingman 1982) and are effective at detecting old (> 50 generations ago) and/or severe (100‐fold change in population size for either contractions or expansions) demographic changes. However, recent declines (within the last 10 generations) are not robustly detected using these methods (e.g., Girod et al. 2011).

A different and commonly utilised alternative is provided by the software BOTTLENECK (Cornuet and Luikart 1996), which provides a more suitable approach for microsatellite genotype datasets where recent population history is being investigated. BOTTLENECK compares the expected heterozygosity (*H*_e,eq_) at mutation–drift equilibrium, based on the observed number of alleles (*k*) among n samples, to the actual (observed) value of expected heterozygosity (*H*_e_) for the samples—allowing detection of recent (within between 2 *N*_e_ and 4 *N*_e_ generations ago) changes in population size based on temporary excess *H*_e_ (*H*_e_ > *H*_e,eq_), resulting from the faster loss of allelic richness than heterozygosity in bottlenecks (Nei, Maruyama, and Chakraborty 1975; Cornuet and Luikart 1996). The same computational approach can also identify heterozygosity deficiency (i.e., *H*_e_ < equilibrium *H*_e,eq_), indicative of recent population expansion (empirically validated by, e.g., Donnelly, Licht, and Lehmann 2001).

### 1.3 The Influence of Genetic Structure on Estimates

A plethora of population genetic analyses can be influenced by unaccounted genetic structure within a dataset, leading to erroneous signals or estimates, due to the common assumption of an idealised population such as the Wright–Fisher model (Fisher 1930; Wright 1931). Likewise, bottleneck detection and *N*_e_ estimates can also be biased by unaccounted population structure (Luikart and Cornuet 1998; Chikhi et al. 2010; Kopatz et al. 2017). Unaccounted spatial genetic structure (Wahlund effects) has been shown to cause large downward biases in the estimation of *N*_e_ using the LD method (Neel et al. 2013; Kopatz et al. 2017; Mergeay et al. 2024). This is due to the impacts of nonrandom mating (i.e., population subdivision) on population LD (England, Luikart, and Waples 2010; Waples and England 2011). This results in cases where global *N*_e_ estimates are considerably lower than the sum of subpopulation *N*_e_ estimates, although large‐scale empirical studies of this interaction are still relatively rare. This has been empirically found for the recovering populations of brown bears ( *Ursus arctos* ) in Finland (Kopatz et al. 2017) and grey wolves ( *Canis lupus* ) on the Iberian peninsula (Mergeay et al. 2024). Furthermore, Kopatz et al. (2017) found that including admixed individuals strongly increased *N*_e_ estimates and suggested more work was needed on their potential high influence in estimating *N*_e_ as well as number of breeders (*N*_b_) through further studies in other species and systems. It follows, therefore, that understanding the genetic structure present in a dataset is important to help ensure estimates are reliable.

### 1.4 Eurasian Otters in the United Kingdom as a Study System to Investigate Signals of Past Population Crashes and Subsequent Expansions

Many populations of large carnivores underwent major declines in the 19th and 20th centuries and are currently showing population expansions, as anthropogenic pressures have been eased through legal protection (Chapron et al. 2014). While such population size increases are positive for conservation, it is important that census population increases in isolation are not regarded as indicating a successful recovery (Thomas et al. 2022a). The persecution of Eurasian otters ( *Lutra lutra* ) in the United Kingdom likely began as far back as the Middle Ages (Lovegrove 2007). Historic records indicate a steady decline in numbers from the 18th century onwards due to anthropogenic predator control, sport hunting and pollution (Jefferies 1989). However, it was not until the 1950s that hunting records showed a sudden and rapid decline in otter numbers, with southern England the most severely affected area. The decline was parallel to that seen in predatory bird populations which suggested that the insecticide dieldrin, along with other organochlorine chemicals, was the cause (Chanin and Jefferies 1978). Dieldrin was introduced in the 1950s as a sheep dip and seed coating and was subsequently detected in 81% of otters examined between 1963 and 1973 (Mason et al. 1986). Voluntary restrictions were placed on the chemical in the 1960s–1970s followed by a mandatory ban in the 1980s.

As a response to the dramatic population decline in otters, systematic national surveys were set up in Wales, England and Scotland, with the first undertaken in the late 1970s (Crawford et al. 1979; Green and Green 1980; Lenton, Chanin, and Jefferies 1980). Successive national surveys for otters in both Wales and England have shown a steady increase in detection of positive signs for otters at survey sites (Crawford 2010; Strachan 2015—see Figure S1 in Thomas et al. 2022a). However, although more frequent and spatially widespread detection of signs, such as otter spraint, indicates that otters have now returned to previously extirpated areas, it is impossible to estimate the change in population size with any degree of certainty (Sainsbury et al. 2019; Mathews et al. 2018). For instance, the number of otters per km of river across a United Kingdom‐wide scale will show high spatial variance. Similarly, regional variation in the degree and rate of otter population declines and subsequent recoveries, along with the heterogeneous landscape and prey availability (Mathews et al. 2018), imply that any estimation of current or past population sizes from national survey data should be treated with extreme caution.

Despite recent population re‐expansion, significant genetic structure persists in the UK otter metapopulation (Hobbs et al. 2011; Stanton et al. 2014; Thomas et al. 2022a; du Plessis et al. 2023a), which broadly reflects expansion from four ‘strongholds’ in (1) Scotland and North England, (2) Wales, (3) South West England and (4) East England. This suggests that re‐establishment of contact between previously isolated subpopulations has not yet resulted in genotypic homogenisation.

The combined wealth of knowledge from national otter surveys and genetic/genomic studies has provided an unusually well‐known account of the history of Eurasian otters in the United Kingdom, along with detailed knowledge of population genetic structure. This background renders UK otters a particularly suitable model system to study the genetic effects of recent population bottlenecks, and to reliably estimate *N*_e_. This setting also provides an excellent opportunity to explicitly evaluate the biases arising from cryptic population structure and admixture on genetic estimates of past demography. We here used a previously published dataset of 407 UK otters from across the United Kingdom spanning 21 years (1994–2014), genotyped at 15 polymorphic microsatellite loci (Thomas et al. 2022a) (more details on the dataset are given below in the Methods section). That study reported (1) the absence of significant increase in genetic diversity over time, and (2) a slow increase in gene flow over time, albeit not enough to lead to a significant reduction in population genetic structuring over time. Based on this dataset, we investigated spatio‐temporal patterns of genetic signals of changes in *N*_e_, with particular attention to any biases arising from admixture, Wahlund effects and temporal lumping of samples across cohorts.

Overall, we expected that bottleneck tests and temporal *N*_e_ comparisons would show signals of past otter population declines. Alternatively, recent and ongoing population expansion in the United Kingdom might override these signals. We expected that detection of either process (decline or expansion) would be sensitive both to spatial variation across the United Kingdom in the degree of population recovery (Thomas et al. 2022a) and differences in methodological/statistical approach. Firstly, we hypothesised that signals from population expansion from BOTTLENECK would be greater in demes which showed the fastest demographic recoveries (rapidity of change), that is, the South West England and Wales regions. Second, we hypothesised to obtain the clearest BOTTLENECK signals of a bottleneck in the area suggested by national otter surveys to have experienced the most severe population decline and to have been the slowest to recover (severity of change), that is, East England. Third, we hypothesised that the *N*_e_ estimate from LDNe for the whole dataset would be less than the sum of the estimates of *N*_e_ for the regional subpopulations due to the presence of admixture LD in the first dataset but not the second. Fourth, we hypothesised that the inclusion of genetically admixed individuals would increase subpopulation *N*_e_ compared to when they are excluded from estimates. Finally, we hypothesised LDNe would yield increasing estimates of *N*_e_ at later time points during the population expansion, mirroring the otter survey results revealing increasing presence of otter signs.

## 2 Methods

### 2.1 Samples, Genotyping and Dataset Production

**FIGURE 1:**
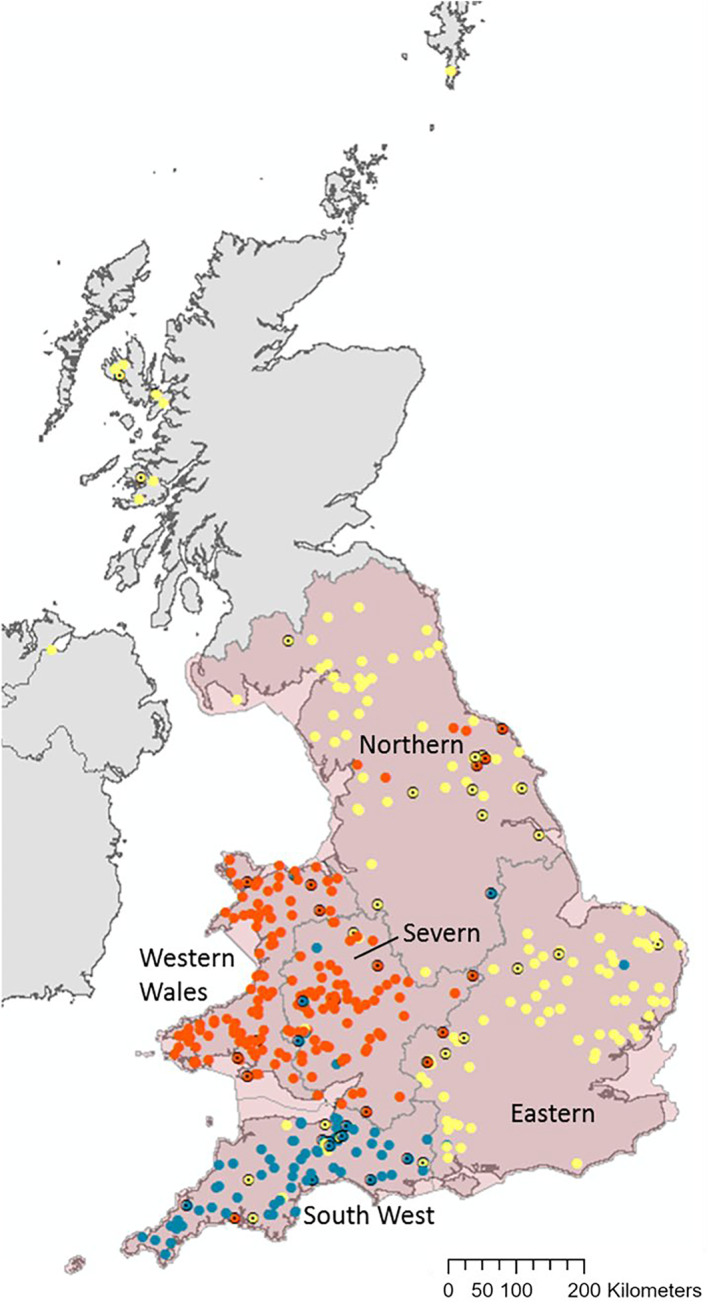
Geographic locations of the 407 genotyped individuals. The five focal River Basin District (RBD) regions of our study are outlined, and shaded in light red. Assignment by STRUCTURE to a genetic cluster at *K* = 3 is denoted by the following colours; orange—Wales and Borders, blue—Southwest, yellow—central and Northern England. A black outline with a black dot in the centre indicates that the individual had a membership value *q* < 0.8 to the given cluster and was therefore classified as ‘admixed’.

We used a georeferenced dataset (Thomas et al. 2022a, 2022b) of 407 muscle tissue samples (Figure 1) from predominantly road‐killed otters held in the Cardiff University Otter Project archive. Based on sampling location, each sample was allocated to a River Basin District (RBD) (Water Framework Directive Cycle 2, Environment Agency 2015; Natural Resources Wales 2015), corresponding to watershed‐based groupings of river catchments.

Host DNA was extracted and genotyped using 15 microsatellite loci (see Thomas et al. 2022a for methods on DNA extraction and microsatellite genotyping). The average number of alleles across all 15 microsatellite loci was 8.2 (range: 6–11) alleles per locus. The average expected and observed heterozygosity across all loci were 0.68 and 0.55, respectively, ranging from 0.46 to 0.77 for expected heterozygosity and 0.40 to 0.60 for observed heterozygosity per locus. Genetic diversity across the spatio‐temporal elements of the dataset showed no significant difference in genetic diversity between years, but significant differences in genetic diversity across space with the Eastern regions of the study area (Eastern and Northern RBDs) being significantly more diverse than the Western regions (Severn, South‐West and Western Wales RBDs) (Thomas et al. 2022a). The samples were collected between 1993 and 2014 from across the United Kingdom, although sampling was sparse and temporally restricted in Scotland and Northern Ireland, meaning some analyses could only be conducted on samples from Wales and England. Otters sampled were predominantly sexually mature adults (58%), with 35% adult size but not yet mature (based on reproductive indicators) and 5% juvenile (dependent young). Previous cementum analysis suggests a small age range (Sherrard‐Smith and Chadwick 2010) with most individuals less than 3 years old. Admixed individuals in the dataset were identified using a membership value *q* < 0.8 from the STRUCTURE (Pritchard, Stephens, and Donnelly 2000) outputs published by Thomas et al. (2022a, 2022b). The cutoff of 0.8 represents a compromise between false‐positive and false‐negative assessment of admixture (Sanchez‐Donoso et al. 2014). The cluster assignment at *K* = 3 was chosen for this purpose as it had strong support using a combination of (i) the ΔK method (Evanno, Regnaut, and Goudet 2005), (ii) likelihood of K (Pritchard and Wen 2003), (iii) was biologically plausible (Janes et al. 2017), and (iv) in Thomas et al. (2022a, 2022b) captured the overarching genetic structure in the population (Figure 1). This allowed the production of datasets at various spatial and temporal scales, as well as allowing analysis with admixed individuals included and excluded, to test the assumptions and biases of each analysis.

### 2.2 Population Bottleneck Analysis

To test for recent changes in effective population size in the dataset, we used BOTTLENECK v1.2.02 (Piry, Luikart, and Cornuet 1999) which uses allele frequency data to detect recent bottleneck or expansion events (Cornuet and Luikart 1996; Luikart et al. 1998). We used all four tests available in BOTTLENECK: the sign test, standardised differences test, Wilcoxon sign‐rank test and the allele frequency distribution or mode shift indicator, but given that the sign test suffers from low statistical power and the standardised differences test requires at least 20 loci, we focused on the results from the Wilcoxon sign‐rank test and the more qualitative allele frequency distribution. The Wilcoxon sign‐rank test has been shown to have relatively high power in detecting population size changes, and although it can be used with as few as four polymorphic loci and any number of individuals, to achieve this high power of detection, it is recommended to use 10–15 polymorphic loci and 15–40 individuals (Luikart and Cornuet 1998). All data combinations tested had 14 or 15 polymorphic loci, while all had more than 15 individuals and many had more than 40 individuals (22/28 datasets, with those with *N* < 40 limited to the temporally restricted analysis), indicating that there should have been high power in our analyses to detect population bottlenecks or expansions using this test.

We used data available on the mutation processes in human microsatellite DNA sequences (Ellegren 2000) to estimate the frequency of adherence to the stepwise mutation model (SMM) for both dinucleotide and tetranucleotide microsatellites. The frequencies were estimated to be 83.8% and 89.5% for dinucleotide and tetranucleotide microsatellites, respectively. We then applied this estimation to the specific panel of microsatellites used to genotype Eurasian otters in this study based on whether each locus had a dinucleotide or tetranucleotide repeat unit (Table 1). This custom frequency was then used as input data for the two‐phased model (TPM) of mutation in BOTTLENECK. This model is considered more appropriate for microsatellite data than the SMM, as microsatellite mutations predominantly comprise single‐step mutations, with multi‐step changes and other mutations being rarer (Di Rienzo et al. 1994; Ellegren 2000). Additionally, if a BOTTLENECK analysis is run using the strict SMM mutation model and loci deviate even slightly from this, simulations have shown that either bottleneck or expansion signals can be seen even for populations which are at mutation–drift equilibrium (Cornuet and Luikart 1996). In addition to the TPM, we also evaluated the datasets for bottleneck signals using the infinite allele model (IAM), an approach that does not take allele length into account and thus may potentially be more sensitive to recent processes than the TPM and SMM (see Swaegers et al. 2014).

**TABLE 1:** Literature‐based inference of the frequency of stepwise mutations for analysed panel of otter microsatellites.

Microsatellite repeat unit	Frequency of single‐step mutations	Number of loci in otter panel
Dinucleotide	83.8%	4
Tetranucleotide	89.5%	11
% Single‐step mutations in otter panel	88%	15

*Note:* Frequency of stepwise mutation model (SMM): Proportion of single‐step mutations among single‐ and multiple‐step microsatellite mutations surveyed by Ellegren (2000); Number of loci: Number of loci (of the total 15 microsatellites used in this study) that are either dinucleotide or tetranucleotide; Freq SMM in otter panel: Estimated frequency of SMM across our three multiplex panels (Thomas et al. 2022a) based on the number of loci of different repeat units.

Each dataset was run for 1000 iterations using the IAM, and also the TPM, with proportion of single‐step mutations set to 88% and the variance set as either 12 or 30 (one run each). To assess the impact of both underlying genetic structure and admixed individuals on the detection of effective population size change using these methods, the dataset followed a hierarchical selection process (Table 2) where the presence of both genetic structure and admixed individuals in the dataset was systematically and sequentially accounted for. This process was repeated with just the most contemporary samples from 2009 and 2014, and also with early (1993–1995 and 1993–1999) and late (2009–2014 and 2014) datasets from the Wales and Borders (Western Wales and Severn RBD regions), where there was sufficient sampling to allow a temporal comparison (i.e., *N* > 15).

**TABLE 2:** Bottleneck results with and without accounting for geographic structure (Wahlund effects) and admixture.

Dataset	N	Mean *H*_exp_	Possible biases		TPM (88%, 30) Wilcoxon *p*	TPM (88%, 12) Wilcoxon *p*	Mode shift	Detected signal
			Genetic structure	Admixed individuals				
All data	407	0.68	Yes	Yes	0.05	0.02	Normal L‐shaped	Expansion
Wales and England	396	0.68	Yes	Yes	0.05	0.02	Normal L‐shaped	Expansion
	347	0.68	Yes	No	0.05	0.02	Normal L‐shaped	Expansion
Eastern England RBD	74	0.72	Yes	Yes	0.01	0.02	Normal L‐shaped	Bottleneck
	64	0.71	No	No	0.01	0.01	Normal L‐shaped	Bottleneck
Northern England RBD	59	0.70	Yes	Yes	ns	ns	Normal L‐shaped	Stable
	42	0.69	No	No	ns	ns	Normal L‐shaped	Stable
South West England RBD	77	0.57	Yes	Yes	0.05	0.02	Normal L‐shaped	Expansion
	58	0.57	No	No	0.08	0.04	Normal L‐shaped	Expansion^a^
Severn RBD	84	0.56	Yes	Yes	0.008	0.002	Normal L‐shaped	Expansion
	71	0.55	No	No	0.01	0.004	Normal L‐shaped	Expansion
Western Wales RBD	102	0.54	Yes	Yes	ns	ns	Normal L‐shaped	Stable
	95	0.54	No	No	ns	ns	Normal L‐shaped	Stable
Central England cluster	132	0.73	No	Yes	ns	ns	Normal L‐shaped	Stable
	112	0.73	No	No	ns	ns	Normal L‐shaped	Stable
South West England cluster	78	0.60	No	Yes	ns	ns	Normal L‐shaped	Stable
	65	0.59	No	No	ns	ns	Normal L‐shaped	Stable
Wales & Borders cluster	186	0.58	No	Yes	0.002	0.001	Normal L‐shaped	Expansion
	170	0.56	No	No	0.01	0.005	Normal L‐shaped	Expansion

*Note:* ‘*Dataset*’ describes the study area, this being either all data, Wales and England, areas defined by River Basin District (RBD), or by genetic cluster (see Figure 1 and explanatory methods). For each subset, two rows of results are presented, these being the results where biases are included, or accounted for, with respect to *Genetic structure* (yes, present in the dataset; no, has been accounted for) and *Admixed individuals* (yes, admixed individuals based on 15 microsatellite loci and a STRUCTURE *q*‐value threshold of 0.8, included in the dataset; no, admixed individuals excluded). *N* is the number of individuals analysed, Mean *H*_exp_ is the mean heterozygosity across samples within each grouping. *TPM* (88%, 30) and *TPM* (88%, 12) Wilcoxon *p*‐values are the *p*‐value for the relevant one‐tailed test using the two‐phase model of mutation with 88% stepwise mutations and variance of 12 or 30, respectively; ns indicates *p*‐value failed to meet the standard alpha value for significance (i.e., *p* **>** 0.05), and underlined *p*‐values show marginally significant values, where the second model was significant. *Mode Shift* is the BOTTLENECK‐classified distribution of allele frequencies; *Detected Signal* describes the population state according to the Wilcoxon test. Note that the IAM yielded significant bottleneck signals for each tested dataset.

^a^ One *p*‐value is significant for expansion, the other *p*‐value (underlined) is only marginally significant for the same signal.

### 2.3 Effective Population Size Estimation

Effective population size (*N*_e_) was estimated using the LD (LDNe) method (Waples and Do 2008) as implemented in NeEstimator v2.1 (Do et al. 2014). LDNe uses the Burrows method to estimate LD with a correction factor to account for using unlinked loci such as microsatellites (Waples 2006). The *N*_e_ estimated by LDNe is thought to quantify *N*_e_ of the recent past, that is, few generations back in time (Ryman, Laikre, and Hössjer 2019; Waples 2023). Especially since our 21‐year sampling allowed us to split the dataset into time periods and geographic regions, LDNe provides an excellent opportunity to look for spatio‐temporal trends. We excluded rare alleles which can upwardly bias *N*_e_ estimates using the *P*_crit_ function (Waples and Do 2010) based on the formula 1/(2 × *N*) < *P*_crit_ < 1/*N* which highlighted that different values of *P*_crit_ were appropriate for different datasets because sample size varied by at least an order of magnitude. Confidence intervals were determined using the jackknife method, which has been shown to perform better than parametric methods (Waples and Do 2008). When reporting the sum of independently estimated *N*_e_ values (e.g., for local demes or temporal subsets), we also report the sum of the lower and upper limits of the 95% confidence intervals, respectively.

We estimated *N*_e_ with and without accounting for both the underlying genetic structure in the dataset and the presence of admixed individuals. Unaccounted genetic structure can lead to admixture LD (England, Luikart, and Waples 2010; Waples and England 2011) and bias *N*_e_ by combining more than one gene pool in the analysis, either upwards or downwards (Kopatz et al. 2017).

Estimates of *N*_e_ for RBD regions and genetic clusters were repeated using only data from 2009 and 2014 to calculate the most contemporary figures across Wales and England, both to compare with those calculated from the last national survey data (Sainsbury et al. 2019; Mathews et al. 2018) and to see if these differed significantly from estimates made using the whole temporal spread of the data. In addition, to these ‘late’ estimates of *N*_e_, a set of ‘early’ estimates were computed for the genetic clusters and RBD regions including samples collected up to 2004. This allowed comparison of the estimated *N*_e_ at two different time points during the population expansion.

For comparison with population size estimates derived from national survey data, a value for the census population size (*N*_c_) was needed, not *N*_e_ as calculated by LDNe. Frankham (1995b) conducted a review of *N*_e_/*N*_c_ ratios using data from 192 species and determined that broadly *N*_e_ was likely to be 0.10–0.11 of N, therefore we used this ratio to put the effective *N*_e_ estimates in context of the national survey population estimates.

## 3 Results

### 3.1 Population Bottleneck Analyses

The IAM yielded significant (*p* < 0.05) heterozygote deficit, that is, a bottleneck signal, for each dataset explored, that is, all 28 spatial and temporal groupings. All results reported below refer to the TPM.

The results from BOTTLENECK from the whole dataset (1993–2014) when population genetic structure was not accounted for (i.e., with the dataset being analysed as one panmictic population) found significant deficiency of actual *H*_e_ compared with equilibrium expectations based on the observed number of alleles (*H*_e,eq_), typically interpreted as signals of a population expansion. However, once the dataset was split geographically into the RBD regions to account for the genetic structure present (circumventing Wahlund effects), many of these apparent expansion signals disappeared (Table 2; for full results, see Table S1). The Eastern RBD region showed a significant bottleneck signal whether admixed individuals were included or not, while the Severn RBD region showed significant expansion. The South West England RBD region had a significant signal of expansion when admixed individuals were included, which became marginal once these were excluded. In contrast, the Northern England and Western Wales RBD regions both showed only signatures of population stability.

Analysis by genetic cluster, as an alternative to grouping individuals by geography (Table 2; Table S1), indicated a significant expansion for the Wales and Borders cluster, while both the Central England and South West England clusters gave signals consistent with stable populations whether admixed individuals were included or not. None of the datasets showed any mode shift in allele frequencies.

Analysis of the most contemporary data (from 2009 and 2014) showed broadly similar patterns to the full dataset (Table 3). The Eastern RBD region showed a weaker bottleneck signal, whereas the South West England RBD region showed an increased signal of expansion and the Severn RBD region changed from an expansion to a stable population signal. Both Northern and Western Wales RBD regions continued to show signals of population stability (for full results, see Table S2).

**TABLE 3:** Bottleneck results from temporal sampling within River Basin District (RBD) regions.

Dataset	Years	*N*	Mean *H*_exp_	*p*	Detected signal
Eastern England RBD region	2009–2014	43	0.71	0.05/0.05	Bottleneck
Northern England RBD region	2009–2014	20	0.69	ns	Stable
South West England RBD region	2009–2014	37	0.58	0.02/0.01	Expansion
Severn RBD region	2009–2014	22	0.54	ns	Stable
Western Wales RBD region	2009–2014	31	0.56	ns	Stable
Wales and Borders cluster	1993–1995	25	0.50	ns	Stable
Wales and Borders cluster	2014	28	0.53	ns	Stable
Wales and Borders cluster	1993–1999	59	0.52	ns	Stable
Wales and Borders cluster	2009–2014	53	0.56	0.02/0.02	Expansion

*Note: N*, number of individuals included in the analysis; Mean *H*_exp_, the mean heterozygosity across samples in the dataset; *p*‐values: The *p*‐values for the relevant one‐tailed Wilcoxon test using the two‐phase model of mutation with 88% stepwise mutation and variance 30 and 12, respectively; detected signal: Population state according to the Wilcoxon test. ns indicates *p*‐value was nonsignificant. Note that the IAM yielded significant bottleneck signals for each tested dataset.

The results from temporal sampling of the Wales and Borders region genetic cluster showed that neither the earliest (1993–1995) nor the latest (2014) samples showed a significant signal of bottleneck or population expansion, and thus the population at both timepoints appeared stable (Table 3). Given the relatively small sample size at these two time points (*N* = 25 and *N* = 28, respectively), we repeated the analysis using a broader timescale of 1993–1999 and 2009–2014. In this analysis, the 1993–1999 dataset showed no signal of recent population size change, but the 2009–2014 dataset had a significant signal for population expansion (for full results, see Table S3).

### 3.2 Effects of Population Structure and Admixture on *N*_e_ Estimates

**FIGURE 2:**
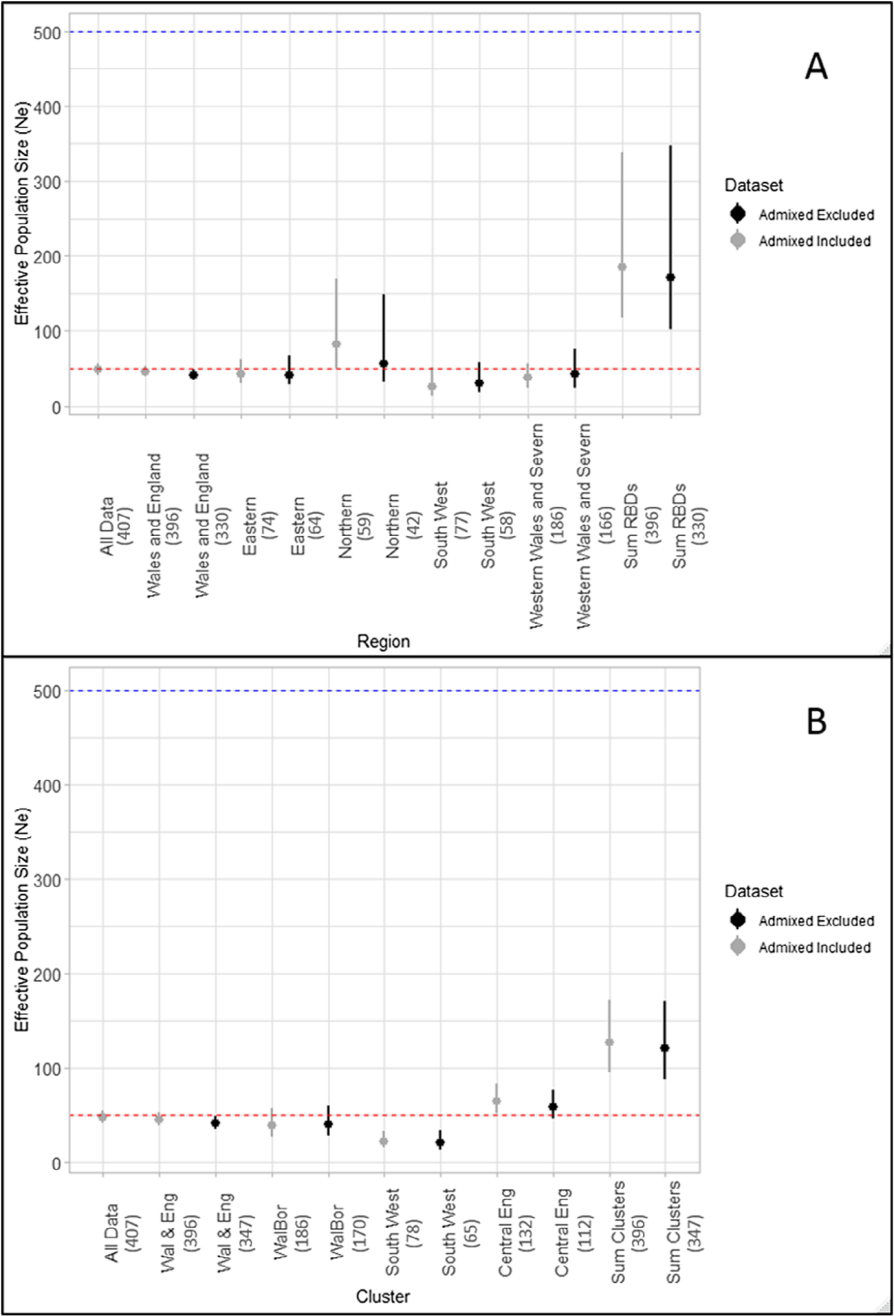
Impact of spatial genetic structuring and admixture on estimates of effective population size (*N*_e_) of UK otters based on single‐sample linkage disequilibrium (LD) methods. Confidence intervals (95%) are based on the jackknife across samples method. Numbers in brackets indicate sample size. Horizontal dashed lines indicate two critical values of *N*_e_ (as proposed by Franklin 1980): 50 in red, to reduce the risk of inbreeding depression; and 500 in blue, to maintain adaptive potential. (A) analysis performed on a geographic basis using River Basin District regions to split the data into populations; (B) analysis performed on a genetic cluster basis using average assignment across 10 STRUCTURE runs at *K* = 3. In both plots, ‘All Data’ and ‘Wales and England’ or ‘Wal & Eng’ refer to the datasets run without consideration of genetic structure. Grey indicates analyses where admixed individuals were included and black indicates analyses where admixed individuals were excluded on a *q* < 0.8 basis.

Estimates of *N*_e_ obtained from tests without accounting for population genetic structuring (i.e., all data combined, and all Wales and England data treated as one population) resulted in *N*_e_ values that were considerably lower than when population structure was accounted for (lower by a factor of approximately 3–4 times than for summed genetic clusters or summed RBD regions, respectively; Figure 2). Excluding admixed individuals from the datasets resulted in different outcomes for different regions. For example, with admixed individuals excluded the estimate of *N*_e_ for the Northern RBD region was lower than with admixed individuals included, whereas for both the South West England and the Severn and Western Wales RBD regions, the opposite was true (Figure 2A). *N*_e_ estimates by genetic cluster varied less when admixed individuals were removed than by RBD region (Figure 2B). In all cases, the 95% confidence intervals of the estimates both with and without admixed individuals overlapped, indicating no significant difference in the estimates. Regardless of the inclusion of admixed individuals, all estimates of *N*_e_ were low with the sum of the RBD region results totalling 170.6 (95% CI: 102.1–348.3), and the sum of the genetic clusters totalling 121.3 (95% CI: 88.4–171.2, see Table S4).

### 3.3 Temporal Changes to Effective Population Size Estimates

**FIGURE 3:**
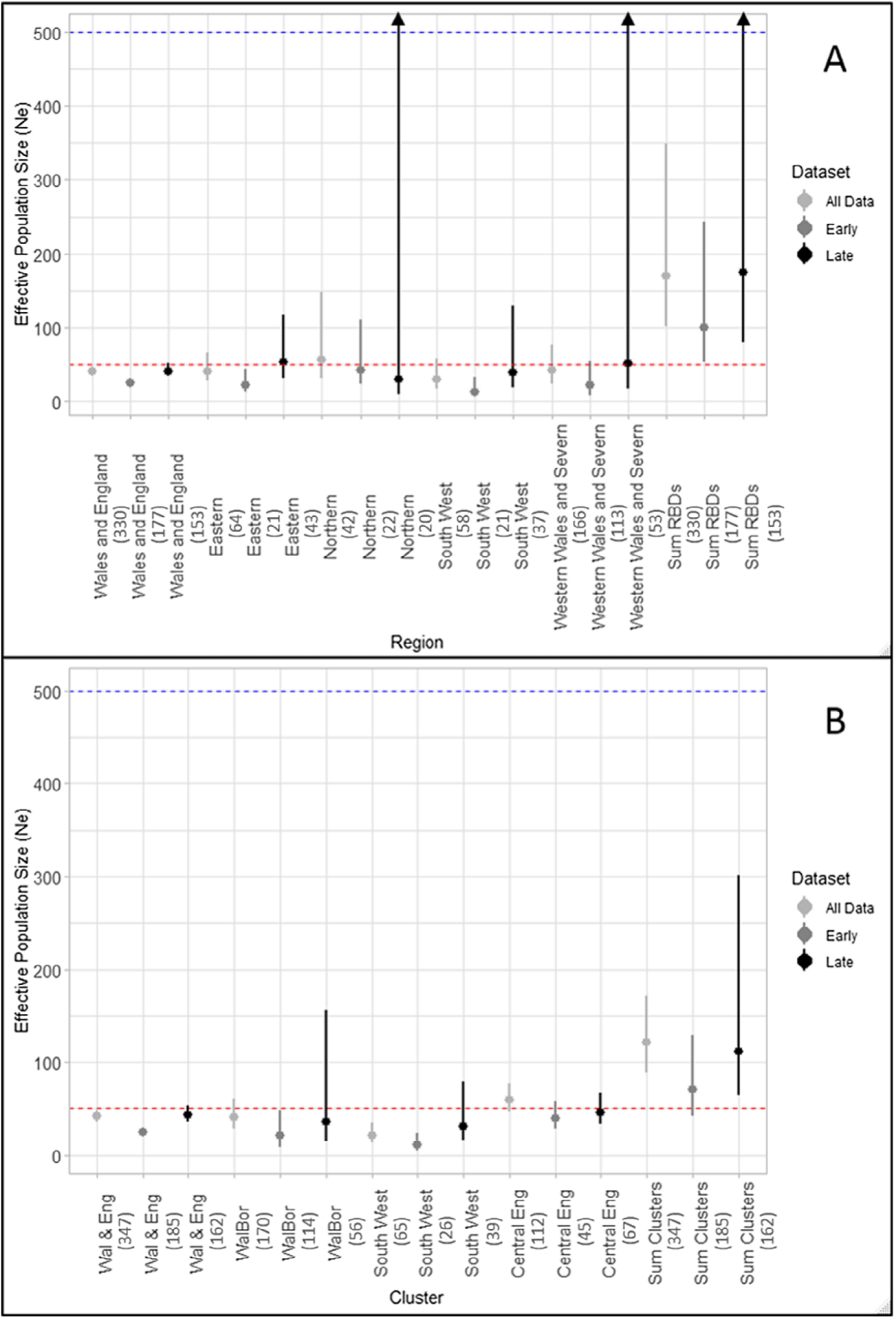
Temporal changes in effective population size (*N*_e_) in UK otters based on single‐sample linkage disequilibrium (LD) methods. Confidence intervals (95%) are based on the jackknife across samples method. Numbers in brackets indicate sample size. Horizontal dashed lines indicate two critical values of *N*_e_ (as proposed by Franklin 1980): 50 in red, to reduce the risk of inbreeding depression; and 500 in blue, to maintain adaptive potential. (A) Analysis performed on a geographic basis using River Basin District regions to split the data into populations; and (B) analysis performed on a genetic cluster basis using average assignment across 10 STRUCTURE runs at *K* = 3. Black arrows indicate cases where the upper 95% confidence interval was estimated to be infinity.

For both RBD regions and genetic clusters, most *N*_e_ estimates increased when using the ‘late’ dataset compared with the ‘early’ dataset (Figure 3 and Table S5). The one exception was the Northern RBD region, where the point estimate for the late dataset was lower than the earlier one, however, the upper confidence interval for this estimate tended to infinity indicating low reliability. Cluster‐based estimates had narrower confidence intervals than RBD region‐based estimates, with *N*_e_ values falling below *N*_e_ = 50 for all three genetic clusters although confidence intervals overlapped this minimum viable population (MVP) boundary. None of the *N*_e_ estimates, including the sum of the cluster estimates, crossed the *N*_e_ = 500 thresholds to maintain long‐term adaptive potential. The more contemporary estimates tended to have lower precision than their respective early data or all data counterparts, as illustrated by wider confidence intervals. The sum of both RBD region and genetic cluster estimates for *N*_e_ was significantly larger than estimates obtained for the whole population (i.e., without consideration of spatial genetic structure) across all temporal groupings. The summed estimates of *N*_e_ were approximately four times (4.0–4.2) larger for the RBD regions and nearly three times (2.6–2.9) larger for the genetic clusters, additionally the 95% confidence intervals for all data versus summed data estimates did not overlap.

## 4 Discussion

Understanding the recent demographic history and current status of populations is critical for their evidence‐based conservation and management. Here, we used an empirical dataset (407 otters sampled across the United Kingdom and 21 years from 1993 to 2014, genotyped at 15 microsatellite loci) to test theoretical expectations associated with the effect of admixture, substructure and sample size on the inference of past population size change and estimates of effective population size. Our findings also provide spatially explicit insights into dynamic changes in genetic variability in this recovering and expanding population—and as such inform about the effectiveness of past management decisions and conservation actions.

### 4.1 Detection of Population Bottlenecks

Bottleneck detection was method dependent. When using the IAM, we obtained bottleneck signals for each of the 28 spatiotemporal groupings (i.e., for each dataset) tested, whereas our two‐phase model (TPM) results were more conservative and suggested that the Eastern England RBD region was the only region showing consistent and significant microsatellite signals of a recent population bottleneck (Table 4). Ubiquitous signals of bottlenecks in UK otters are generally plausible based on national survey data and genomic analyses (Table 4). One exception to this is North England and Scotland, where genomic data indicated a slow, long‐term decline over past centuries and no recent population growth. Survey data suggested that the Scottish population remained at higher densities and more stable than otters elsewhere in the United Kingdom with presence of otter signs never decreasing below 58% of survey sites (Findlay, Alexander, and Macleod 2015), hence genomic detection of bottleneck in northern England may have been obscured by the amalgamation of North England and Scotland samples by du Plessis et al. (2023a), while in this current study, Scotland was not included in the ‘North’ grouping. The widespread bottleneck signals in UK otters likely stem from the combination of two factors which have shaped the stronghold populations historically: firstly, the reduction of local *N*_e_ from population declines, and secondly, from disconnection of demes (i.e., reduction of gene flow) at the metapopulation level as the stronghold populations became more isolated from each other, which has been shown to create genetic bottleneck signatures even in the absence of demographic declines (Broquet et al. 2010).

**TABLE 4:** Comparison of demographic history and detected signals of population bottlenecks and expansions across different studies of Eurasian otters ( *Lutra lutra* ) in the United Kingdom.

RBD region	Current Study		Genomic signals in du Plessis et al. 2023a	National otter surveys 1977–2010
	TPM signal	IAM signal		
Eastern	Bottleneck	Bottleneck	Nadir of bottleneck indicates a *N*_e_ of well below 50, and likely below 10, followed by partial recovery to a *N*_e_ of around 50^a^	Area with lowest presence of otter sign in early surveys and slowest increase in otter sign
Northern	Stable	Bottleneck	Weak but long‐term population decline shown, with no bottleneck (note: sample included Scottish individuals, not only Northern England)	Large increase in % of survey sites positive for otter signs across survey period, but surveys in Scotland (contiguous to Northern RBD) show there was no large‐scale decline in signs there
Severn	Expansion	Bottleneck	Nadir of bottleneck indicates an *N*_e_ of over 100 with subsequent partial recovery (note: dataset is across all of Wales; see ‘Western Wales’ detailed below)	Large increase in % of survey sites positive for otter sign across survey period
South West	Expansion	Bottleneck	Nadir of bottleneck indicates a *N*_e_ of around 50 with subsequent ~10‐fold increase in *N*_e_	Large increase in % of survey sites positive for otter signs across survey period
Western Wales	Stable	Bottleneck	Nadir of bottleneck indicates a *N*_e_ of over 100 with subsequent approximate doubling of *N*_e_ (note: dataset is across all of Wales; see ‘Severn’ detailed above)	Stronghold area, with % otter sign staying higher than across rest of Wales and England, and increasing earlier

*Note:* ‘Current study’ indicates the BOTTLENECK analyses carried out in this study using microsatellite data; ‘Genomic signals’ indicates a genomic dataset analysed by du Plessis et al. 2023a, (using GONE [Santiago et al. 2020]), and ‘National Otter Survey data 1977–2010’ indicates five national surveys carried out across Wales, England and Scotland between 1977 and 2010 recording the presence of otter sign (Crawford 2010; Findlay, Alexander, and Macleod 2015; Strachan 2015).

^a^ Note that Eastern England otters appear impacted by introgression of alleles from Eurasian otters from Asia, both for autosomal and mitochondrial loci (du Plessis et al. 2023a, 2023b).

Our two‐phase model (TPM) results provide a heterogeneous picture about the population history of UK otters (Table 4). Availability of both national survey data and conclusions of a genomic study allowed us to provide detailed context, exploring the plausibility and limitations of BOTTLENECK outcomes (Table 4). This comparison indicates (1) that the most extreme population bottleneck, which occurred in the Eastern RBD region, was detected in our BOTTLENECK analyses (IAM and TPM). (2) We also detected TPM‐based population expansions in areas where the recovery was most pronounced (South West and Severn RBD regions). (3) Absence of significant signals of population size change from TPM‐based BOTTLENECK analyses for Northern and Western Wales RBDs were consistent with genomic *N*_e_ estimates from du Plessis et al. (2023a) remaining high (Northern) or over 100 (Western Wales), and broadly consistent with national survey data. However, the Northern area did show a large increase in percentage of otter survey sites that were positive for otter signs between 1977 and 2010. It is possible that significant population growth did occur in North England, while the contiguous Scottish otter population (despite showing genomic evidence of long‐term declining *N*_e_) did not experience any large‐scale declines in otter sign (Findlay, Alexander, and Macleod 2015).

Overall, this indicates that the microsatellite dataset when analysed using BOTTLENECK with the TPM detected the population changes that may be considered most important for conservation management: Population bottlenecks were detected where the genomic data showed that *N*_e_ had reduced below 50 and had not subsequently recovered to above this minimum population viability figure, and population expansions were detected where genomic data showed that *N*_e_ had made multiple‐fold recovery, especially where this was from a minimum *N*_e_ of below 50.

Our results, whether based on IAM or TPM, differ from those previously found in a pan‐European study (Randi et al. 2003) which included samples from the United Kingdom and did not detect any sign of population bottlenecks. However, the sample sizes per country in that study were relatively limited (ranging from three to 29, with only five samples from the United Kingdom), largely below the threshold advised by Cornuet and Luikart (1996) for analysis with BOTTLENECK. A previous genetic study of otters in the United Kingdom by Hobbs et al. (2011) also found no evidence of population bottlenecks, apart from one subpopulation in Northern England and the Scottish Borders region. These differences might reflect the more recent sampling in the current study and be indicative of temporal change, going from bottlenecks/stasis to population expansion. However, a more likely explanation is the different approach to assignment of individuals into subpopulations. Hobbs et al. (2011) tested 11 subpopulations across the United Kingdom designated through progressive partitioning analysis (successive splitting of genetic datasets into the two most genetically differentiated groups), whereas the current study focused on three overarching genetic clusters and four RBD regions as designation of subpopulations.

We note that the IAM yielded a different picture of recent demographic history than the TPM. One possible explanation could be that the two models provide a different temporal perspective of demographic history and/or show different sensitivity to admixture (Swaegers et al. 2014): The IAM does not model the genetic distance between different alleles, and may therefore show more recent processes than the TPM (and SMM), which incorporate evolutionary history of alleles further back in time. Especially in the face of immigration from populations with divergent microsatellite alleles, gaps in allele size distribution will require the TPM/SMM to model numerous mutational steps. Under the TPM/SMM, immigration from divergent populations could therefore lead to false bottleneck signals, in fact manifesting as deviation from migration–drift equilibrium that are mistaken as deviation from mutation–drift equilibrium. If immigration were a major driver of population‐level variation in our dataset, we would therefore expect to see *more* deviations from equilibrium with the TPM than the IAM. We, however, observe the opposite, with the IAM‐based analyses yielding deviation from equilibrium for each of the 28 tested datasets. Given that UK otter populations appear to have gone through both declines and subsequent re‐expansions, it is possible that the IAM and TPM capture slightly different aspects of these processes.

As predicted, when population genetic structure was not accounted for in the dataset, the TPM indicated that there were signals of population expansion. It is likely that this signal was the result of the violation of assumptions, rather than a genuine signal since when the regions were analysed separately, and admixed individuals were removed from the regional datasets, there was an increase in the significance of the Wilcoxon's tests in the case of population bottlenecks and a decrease in the significance of population expansions. Our spatio‐temporal dataset of otter genotypes from the UK stronghold populations therefore reinforces previous suggestions that both spatial (Wahlund) effects and admixture LD have impacts on linkage disequilibrium patterns, which can bias inferences of past population demography and current *N*_e_ (Waples and England 2011).

Notably, results from BOTTLENECK are restricted in the available outcome, only being able to show evidence of either a bottleneck, an expansion or an absence of significant evidence. Hence, when population history is more complex than a single period of demographic change (such as population bottlenecks followed by re‐expansion as for UK otters and many currently recovering species), BOTTLENECK appears likely to pick up a key feature of past demographic history, but more nuanced insights can be provided by approaches such as GONE.

### 4.2 Effective Population Size

As predicted, the *N*_e_ estimates for the datasets without consideration of genetic structure were considerably lower than the estimates based on the sum of either the RBD regions or the genetic clusters. Such an underestimation of *N*_e_ when genetic substructure is not taken into consideration is of similar magnitude to that found by Kopatz et al. (2017), whose estimates were smaller by a factor of nearly 3, when substructure in the dataset was not accounted for (the factors in the present study ranged from ca. 3 to 4, for genetic clusters and RBD regions, respectively).

The inclusion of individuals with admixed genotypes had less predictable effects on the analyses, with some regions or clusters having increased *N*_e_ estimates once admixed individuals were removed (e.g., South West England RBD region), while others yielded decreased *N*_e_ estimates (e.g., Northern England RBD region). This is somewhat in contrast with the findings of Kopatz et al. (2017) who found that the inclusion of admixed individuals caused a large upward bias in *N*_e_ estimates in brown bear populations in Finland. These differences may be because there was less structure in the brown bear population (which had only two subpopulations), potentially resulting in less complex patterns of admixture (Kopatz et al. 2017). Another potential factor could be the relative proportion of admixed individuals in each dataset, but Kopatz et al. (2017) detected a similar proportion (12%) of admixed individuals in their overall dataset as in our dataset. Finally, results by Kopatz et al. (2017) could have a larger effect on admixed individuals than in our study, since they likely included alleles deriving from several adjacent mainland European brown bear populations (see also Kopatz et al. 2021). Similar strong effects of admixture LD on local *N*_e_ estimates have also been found in wolves and frogs (Cox, Neyrinck, and Mergeay 2024; Mergeay et al. 2024), highlighting that spatial scale of the sampling has important consequences on the inferences. Furthermore, recent work has shown that studies utilising a moderate number of microsatellites (such as the current study) may underestimate actual admixture that becomes apparent from genomic‐scale datasets (Gómez‐Sánchez et al. 2018), and indeed du Plessis et al. (2023a) showed that the panel of 15 microsatellites appeared to underestimate admixture among UK otter stronghold populations compared with whole‐genome sequencing data. The effect of admixed individuals on estimates of *N*_e_ may differ between study systems and populations, therefore understanding such potential biases clearly warrants further investigation.

For the estimates using data from all time points, the South West England genetic cluster exhibited the smallest *N*_e_, with both the point estimate and the confidence interval below 50, while the Northern RBD region had the highest *N*_e_, with an upper 95% CI of over 100. This larger population size estimate could be due to the genetic contiguity of Northern English otters with the Scottish population, or the fact that the population in the 1990s was augmented by releases of rehabilitated otters likely from other parts of the United Kingdom (Green 1997). The obtained *N*_e_ estimate for Northern England therefore likely represents an area larger than that which is covered by our sampling.

Census population size estimates derived from national otter survey data across Wales and England give a population estimate of 3900 for the study area (Mathews et al. 2018). The highest estimates from this study (the summed *N*_e_ from across RBD regions with and without admixed individuals) suggest *N*_e_ values of 185.6 and 170.6 individuals, respectively, which, using the 0.1 ratio generalisation of *N*_e_/*N*_c_ (Frankham 1995a, 1995b; Hoban et al. 2021), translates to census population sizes of 1856 and 1706 for Wales and England. This suggests that the census population size estimate of 3900 otters based on national survey results (which is considered to have low reliability due to the methods used) overestimates the true population size—although upper 95% confidence intervals from the current study encompass estimates of up to 3387 and 3483 (with and without admixed individuals, respectively) which are nearer, but still lower than, the estimate calculated by Mathews et al. (2018).

LDNe (as with other single and two‐sample estimators of *N*_e_) assumes discrete generations, an assumption that otter population demography violates. Overlapping generations within a dataset have been shown to produce estimates that are more reflective of the number of breeders than of *N*_e_, but if the number of cohorts sampled is enough to represent a generation, then the estimate will be approximately equal to *N*_e_ (Waples, Antao, and Luikart 2014). Generation time in otters is estimated by Randi et al. (2003) to be 3 years and Pacifici et al. (2013) to be 7.6 years. Therefore, despite the presence of generation overlap in our dataset, due to the number of years covered by the sampling regime, we would expect the estimates to be approximately equal to *N*_e_, although estimates using all of the data time points may be more reliable estimates of *N*_e_ than those using temporal subsampling. However, recent population size change may also have effects on *N*_e_ estimation, by altering the pattern of linkage disequilibrium, which can bias the estimates either upwards or downwards for a few generations (Waples 2005, 2023). Given the population history of otters in the United Kingdom and the results from the BOTTLENECK analyses in our study, our *N*_e_ estimates may be biased due to recent changes in size, further reinforcing the need for continued genetic monitoring of the population.

### 4.3 Minimum Viable Populations (MVPs)

The discussion of what constitutes a MVP has been ongoing in the field of conservation genetics since 1980 when Franklin (1980) first proposed the ‘50/500 rule’. This rule stipulates that to avoid inbreeding depression in the short term, a minimum of *N*_e_ ≥ 50 is required, with a larger minimum of *N*_e_ ≥ 500 required to preserve evolutionary potential and adaptive variation in the long term.

As more studies, especially on wild populations, have accumulated, questions have been raised over whether the two minimum *N*_e_ sizes of 50 and 500 are large enough to avoid detrimental loss of genetic diversity over their respective timeframes. Frankham, Bradshaw, and Brook (2014) proposed that the rule be changed to 100/1000 based on new evidence over the last 30 years, although others maintain that the 50/500 rule is sufficient (Jamieson and Allendorf 2012). The estimates of *N*_e_ for the otter population in Wales and England from this study fall clearly below either of the proposed minimum values (500 or 1000) for long‐term viability, indicating that the evolutionary potential of the population and its ability to adapt to future environmental changes and stressors is currently at risk. In the short term, many of the geographic regions and genetic clusters are estimated to have an *N*_e_ of around 50 indicating that they could also be at risk of inbreeding depression.

The emphasis on genetic diversity in monitoring and management of wild populations has been increasing over past decades (Hoban et al. 2013), with recent renewed calls for genetic monitoring to be included in international policy (Laikre et al. 2020), and subsequent uptake in the Kunming‐Montreal Global Biodiversity Framework (Convention of Biodiversity 2022). While discussions in the scientific community around how best to word the target to policymakers and conservation managers are ongoing (Hoban et al. 2020; Frankham 2021; Laikre et al. 2021), there is broad agreement that an indicator that uses *N*_e_ to track the maintenance of genetic diversity in wild populations and species, as well as domesticated ones, is vital.

Here, we have shown that despite being hailed as a conservation success story (Crawford 2010), otter populations across Wales and England have not yet reached the *N*_e_ necessary for long‐term viability, with many estimates additionally falling within the bounds of questionable viability over the short term. While recent work indicates that gene flow between the stronghold populations is still increasing (Thomas et al. 2022a), the high and maintained F_ST_ values between RBD regions relating to the three main genetic clusters across the study area indicate that substantial genetic structure remains among the former stronghold populations, and hence that genetic recovery is lagging behind the demographic recovery of Eurasian otters in the United Kingdom (Thomas et al. 2022a).

### 4.4 Further Work

Continued genetic monitoring of the otter population in Great Britain is advised, to track *N*_e_ and other genetic diversity metrics, as the population continues to recover. The national otter surveys, which provided a means of monitoring otter presence across the United Kingdom over the last 40 years, are infrequent and are unable to provide robust population estimates (Crawford 2010; Strachan 2015; Kean and Chadwick 2021), leaving a gap in our knowledge of both the current distribution and continued expansion of the otter population. The low *N*_e_ estimates reported in our study indicate the importance of including genetic monitoring of species in national monitoring plans: Importantly, the conclusions drawn from successive national surveys using otter signs (e.g., spraint and footprints), namely, of a robust population close to panmixia, are not supported by the genetic evidence.

A previous study by Stanton et al. (2014) indicated that the most pronounced genetic divide in the otter population of Great Britain was a North–South split, with otters in the area approximately equivalent to the Northern RBD region grouped with those in Scotland. Limitations in the available sample size and temporal coverage of Scotland meant that it was not possible to appropriately investigate this area in the current study. Extending the genetic monitoring to include the Scottish population of otters would not only provide a fuller view of the situation across Great Britain but also resolve whether the Northern RBD region is genetically contiguous with this population, and allow *N*_e_ estimates for this region to be put in more detailed context.

## 5 Conclusions

Cryptic population structure has been discovered in a wide range of highly mobile species with continuous distributions (Sacks, Brown, and Ernest 2004; Pilot et al. 2006), and also for otters in the United Kingdom (Thomas et al. 2022a). Observations of seemingly continuous distributions in these species may result in the incorrect assumption that populations are largely panmictic, therefore genetic data are vital in determining metapopulation structure and the connectivity among subpopulations. Additionally, spatial processes can bias many genetic parameter estimates through violation of the Wright–Fischer idealised population model (Fisher 1930; Wright 1931), and therefore spatial genetic structure can lead to erroneous results when left unaccounted for. Our study adds to previous evidence (e.g., Neel et al. 2013; Kopatz et al. 2017; Mergeay et al. 2024) showing this to be highly relevant when estimating spatio‐temporal changes in *N*_e._ These findings underscore that a comprehensive understanding of population structuring is critical for demographic and genetic monitoring programmes of endangered species.

Despite a well‐documented range expansion and accompanying population expansion over the last 40 years, otters in Wales and England still exhibit small effective population sizes that are well below those required for long‐term viability. Several of the genetic subpopulations and regions also fall below the effective population size required to avoid inbreeding depression and maintain viability in the short term. The South‐East of England, where the population decline was most severe and the population has taken longest to recover, still shows the genetic signature of a population bottleneck, whereas other areas, such as the Severn, have signatures of population expansion. These results paint a more precarious picture than that of the last national surveys in Wales and England which showed otter presence at 90% and 59% of surveyed sites, respectively (Crawford 2010; Strachan 2015), and highlight the need for continued monitoring of the otter population. Small effective population sizes may reduce the ability of the otter subpopulations across Wales and England to respond to future environmental changes and threats as their adaptive potential is reduced.

Monitoring of *N*_e_ in Eurasian otters is particularly important relative to *N*_c_ because observational estimates of *N*_c_ are so methodologically limited: The elusive behaviour of the species limits national surveys to focus on otter signs such as spraint and tracks which are vulnerable to Type II errors (Reid et al. 2013); lack of individual markings largely precludes individual identification in camera footage (Gil‐Sánchez and Antorán‐Pilar 2020); and *N*_c_ estimates are based on presumed home range size, which is likely a highly variable trait (e.g., Ó Néill et al. 2009). Significant improvements in this are not likely. In contrast, estimation of *N*_e_ could be relatively cost‐efficient compared with investment‐improved *N*_c_ estimates, since genetic monitoring could be routinely conducted based on existing collection of otters found dead across the United Kingdom and which are sent to Cardiff University Otter project (as done in the present study).

## Conflicts of Interest

The authors declare no conflicts of interest.

## Supporting information


**Data S1.** Supplementary Information.

## Data Availability

The genotyping data including sample metadata are available on Dryad (Thomas et al. 2022b). Thomas, N. E., Hailer, F., Bruford, M. W., Chadwick, E. A., 2022b. Country‐wide genetic monitoring over 21 years reveals lag in genetic recovery despite spatial connectivity in an expanding carnivore (Eurasian otter, *Lutra lutra*) population [Dataset]. Dryad.https://doi.org/10.5061/dryad.v6wwpzh0h.
