# Supplementary material for: Spatio‐Temporal Changes in Effective Population Size in an Expanding Metapopulation of Eurasian Otters
Source: Evol Appl. 2025 Jan 17;18(1):e70067. doi: 10.1111/eva.70067 (PMC11742082; doi:10.1111/eva.70067)
Supplement: Supplementary file 1 — Data S1. Supplementary Information. [file EVA-18-e70067-s001.docx]

**Supplementary Information for:**

**Spatio-temporal changes in effective population size in an expanding metapopulation of Eurasian otters**

Nia Evelyn Thomas^1*^, Elizabeth A. Chadwick^1^, Michael W. Bruford^1,**^, Frank Hailer^1,2,*^

^1^ Organisms and Environment, School of Biosciences and Water Research Institute, Sir Martin Evans Building, Museum Avenue, Cardiff University, CF10 3AX, Cardiff, Wales, UK.

^2^ Cardiff University-Institute of Zoology Joint Laboratory for Biocomplexity Research (CIBR), Beijing, China

* Corresponding authors: Nia E. Thomas ([nia.e.thomas@gmail.com](mailto:nia.e.thomas@gmail.com)) and Frank Hailer ([HailerF@cardiff.ac.uk](mailto:HailerF@cardiff.ac.uk))

** deceased during the preparation of this manuscript, but had significant input on this study. The authors express their deep sorrow for losing an irreplaceable colleague, who relentlessly spread kindness, enthusiasm, knowledge and inspiration.

ORCiD IDs: Nia Thomas (0000-0003-4412-4286), Elizabeth A Chadwick ([0000-0002-6662-6343](https://orcid.org/0000-0002-6662-6343)), Michael W. Bruford ([0000-0001-6357-6080](https://orcid.org/0000-0001-6357-6080)), Frank Hailer (0000-0002-2340-1726)

### **Table S1: Full Bottleneck results with and without accounting for genetic structure and admixture.** N: number of individuals analysed; Mean He: the mean heterozygosity across samples; Possible biases (genetic structure): Yes indicates genetic structure present in the dataset, No indicates genetic structure accounted for; Possible biases (admixed individuals): Yes indicates admixed individuals (based in data from 15 microsatellite loci and a STRUCTURE q-value threshold of 0.8) were included in the dataset, No indicates admixed individuals excluded from the dataset; Test: the specific test within BOTTLENECK; Test Stat: the test statistic; p-value: p-value of the given test; SMM: single mutation model; TPM (88%, 30): Two-Phase mutation model with 88% stepwise mutations and variance of 30; TPM (88%, 12): Two-Phase mutation model with 88% stepwise mutations and variance of 12; Mode Shift: BOTTLENECK indicator of the distribution of allele frequencies. Significant p-values (p<0.05) are highlighted in bold.

| **Dataset** | **N** | **Mean H_e_** | **Possible biases** | | **Test** | **SMM** | | **TPM (88%, 30)** | | **TPM (88%, 12)** | | **Mode-Shift** |
| --- | --- | --- | --- | --- | --- | --- | --- | --- | --- | --- | --- | --- |
|  |  |  | **Genetic structure** | **Admixed individuals** |  | **Test Stat** | **p- value** | **Test Stat** | **p- value** | **Test Stat** | **p- value** |  |
| All Data | 407 | 0.68 | Yes | Yes | Sign Test (No. loci with heterozygosity excess) | 2 | **<0.001** | 5 | **0.040** | 5 | **0.039** | Normal L-shaped |
|  |  |  |  |  | Standardized differences* | -7.781 | **<0.001** | -2.073 | **0.020** | -2.778 | **0.003** |  |
|  |  |  |  |  | Wilcoxon test (one-tail for deficiency i.e. expansion) |  | **<0.001** |  | **0.047** |  | **0.021** |  |
|  |  |  |  |  | Wilcoxon test (one-tail for excess i.e. bottleneck) |  | 1.000 |  | 0.958 |  | 0.982 |  |
|  |  |  |  |  | Wilcoxon test (two-tail) |  | **<0.001** |  | 0.095 |  | **0.041** |  |
| Wales and England | 396 | 0.68 | Yes | Yes | Sign Test (No. loci with heterozygosity excess) | 2 | **<0.001** | 5 | **0.041** | 5 | **0.044** | Normal L-shaped |
|  |  |  |  |  | Standardized differences* | -8.089 | **<0.001** | -2.083 | **0.019** | -2.996 | **0.001** |  |
|  |  |  |  |  | Wilcoxon test (one-tail for deficiency i.e. expansion) |  | **<0.001** |  | 0.0535 |  | **0.018** |  |
|  |  |  |  |  | Wilcoxon test (one-tail for excess i.e. bottleneck) |  | 1.000 |  | 0.953 |  | 0.985 |  |
|  |  |  |  |  | Wilcoxon test (two-tail) |  | **<0.001** |  | 0.107 |  | **0.040** |  |
| Wales and England | 347 | 0.68 | Yes | No | Sign Test (No. loci with heterozygosity excess) | 3 | **0.002** | 5 | 0.043 | 5 | **0.039** | Normal L-shaped |
|  |  |  |  |  | Standardized differences* | -7.763 | **<0.001** | -2.234 | 0.013 | -2.904 | **0.002** |  |
|  |  |  |  |  | Wilcoxon test (one-tail for deficiency i.e. expansion) |  | **<0.001** |  | **0.047** |  | **0.021** |  |
|  |  |  |  |  | Wilcoxon test (one-tail for excess i.e. bottleneck) |  | 1.000 |  | 0.958 |  | 0.982 |  |
|  |  |  |  |  | Wilcoxon test (two-tail) |  | **0.001** |  | 0.095 |  | **0.041** |  |
| Eastern RBD Region | 74 | 0.72 | No | Yes | Sign Test (No. loci with heterozygosity excess) | 10 | 0.385 | 13 | 0.023 | 13 | **0.023** | Normal L-shaped |
|  |  |  |  |  | Standardized differences* | -0.852 | 0.197 | 1.412 | 0.079 | 1.183 | 0.118 |  |
|  |  |  |  |  | Wilcoxon test (one-tail for deficiency i.e. expansion) |  | 0.849 |  | 0.989 |  | 0.982 |  |
|  |  |  |  |  | Wilcoxon test (one-tail for excess i.e. bottleneck) |  | 0.165 |  | **0.013** |  | **0.021** |  |
|  |  |  |  |  | Wilcoxon test (two-tail) |  | 0.330 |  | **0.026** |  | **0.041** |  |
| Eastern RBD Region | 64 | 0.71 | No | No | Sign Test (No. loci with heterozygosity excess) | 10 | 0.386 | 12 | 0.077 | 13 | **0.024** | Normal L-shaped |
|  |  |  |  |  | Standardized differences* | -0.666 | 0.253 | 1.392 | 0.0820 | 1.113 | 0.133 |  |
|  |  |  |  |  | Wilcoxon test (one-tail for deficiency i.e. expansion) |  | 0.661 |  | 0.991 |  | 0.989 |  |
|  |  |  |  |  | Wilcoxon test (one-tail for excess i.e. bottleneck) |  | 0.360 |  | **0.011** |  | **0.013** |  |
|  |  |  |  |  | Wilcoxon test (two-tail) |  | 0.720 |  | **0.022** |  | **0.026** |  |
| Northern RBD Region | 59 | 0.7 | No | Yes | Sign Test (No. loci with heterozygosity excess) | 5 | 0.0383 | 10 | 0.388 | 10 | 0.385 | Normal L-shaped |
|  |  |  |  |  | Standardized differences* | -2.867 | **0.002** | -0.067 | 0.473 | -0.451 | 0.326 |  |
|  |  |  |  |  | Wilcoxon test (one-tail for deficiency i.e. expansion) |  | **0.047** |  | 0.533 |  | 0.489 |  |
|  |  |  |  |  | Wilcoxon test (one-tail for excess i.e. bottleneck) |  | 0.958 |  | 0.489 |  | 0.533 |  |
|  |  |  |  |  | Wilcoxon test (two-tail) |  | 0.095 |  | 0.978 |  | 0.978 |  |
| Northern RBD Region | 42 | 0.69 | No | No | Sign Test (No. loci with heterozygosity excess) | 7 | 0.230 | 10 | 0.378 | 10 | 0.376 | Normal L-shaped |
|  |  |  |  |  | Standardized differences* | -1.727 | 0.042 | 0.415 | 0.339 | 0.055 | 0.478 |  |
|  |  |  |  |  | Wilcoxon test (one-tail for deficiency i.e. expansion) |  | 0.227 |  | 0.700 |  | 0.598 |  |
|  |  |  |  |  | Wilcoxon test (one-tail for excess i.e. bottleneck) |  | 0.789 |  | 0.319 |  | 0.423 |  |
|  |  |  |  |  | Wilcoxon test (two-tail) |  | 0.454 |  | 0.639 |  | 0.847 |  |
| South West RBD Region | 77 | 0.57 | No | Yes | Sign Test (No. loci with heterozygosity excess) | 2 | **<0.001** | 6 | 0.108 | 6 | 0.101 | Normal L-shaped |
|  |  |  |  |  | Standardized differences* | -4.798 | **<0.001** | -1.909 | **0.028** | -2.227 | **0.013** |  |
|  |  |  |  |  | Wilcoxon test (one-tail for deficiency i.e. expansion) |  | **<0.001** |  | **0.047** |  | **0.024** |  |
|  |  |  |  |  | Wilcoxon test (one-tail for excess i.e. bottleneck) |  | 1.000 |  | 0.958 |  | 0.979 |  |
|  |  |  |  |  | Wilcoxon test (two-tail) |  | **<0.001** |  | 0.095 |  | **0.048** |  |
| South West RBD Region | 58 | 0.57 | No | No | Sign Test (No. loci with heterozygosity excess) | 3 | **0.003** | 6 | 0.125 | 6 | 0.125 | Normal L-shaped |
|  |  |  |  |  | Standardized differences* | -4.16 | **<0.001** | -1.495 | 0.067 | -1.769 | **0.038** |  |
|  |  |  |  |  | Wilcoxon test (one-tail for deficiency i.e. expansion) |  | **<0.001** |  | 0.076 |  | **0.042** |  |
|  |  |  |  |  | Wilcoxon test (one-tail for excess i.e. bottleneck) |  | 1.000 |  | 0.932 |  | 0.964 |  |
|  |  |  |  |  | Wilcoxon test (two-tail) |  | **<0.001** |  | 0.151 |  | 0.083 |  |
| Severn RBD Region | 84 | 0.56 | No | Yes | Sign Test (No. loci with heterozygosity excess) | 1 | **<0.001** | 5 | **0.039** | 4 | **0.011** | Normal L-shaped |
|  |  |  |  |  | Standardized differences* | -5.96 | **<0.001** | -2.8 | **0.003** | -3.236 | **<0.001** |  |
|  |  |  |  |  | Wilcoxon test (one-tail for deficiency i.e. expansion) |  | **<0.001** |  | **0.008** |  | **0.002** |  |
|  |  |  |  |  | Wilcoxon test (one-tail for excess i.e. bottleneck) |  | 1.000 |  | 0.994 |  | 0.999 |  |
|  |  |  |  |  | Wilcoxon test (two-tail) |  | **<0.001** |  | **0.015** |  | **0.003** |  |
| Severn RBD Region | 71 | 0.55 | No | No | Sign Test (No. loci with heterozygosity excess) | 1 | **<0.001** | 4 | **0.011** | 4 | **0.011** | Normal L-shaped |
|  |  |  |  |  | Standardized differences* | -5.405 | **<0.001** | -2.415 | **0.008** | -2.838 | **0.002** |  |
|  |  |  |  |  | Wilcoxon test (one-tail for deficiency i.e. expansion) |  | **<0.001** |  | **0.013** |  | **0.005** |  |
|  |  |  |  |  | Wilcoxon test (one-tail for excess i.e. bottleneck) |  | 1.000 |  | 0.989 |  | 0.996 |  |
|  |  |  |  |  | Wilcoxon test (two-tail) |  | **<0.001** |  | **0.026** |  | **0.010** |  |
| Western Wales RBD Region | 102 | 0.54 | No | Yes | Sign Test (No. loci with heterozygosity excess) | 2 | **<0.001** | 7 | 0.248 | 7 | 0.247 | Normal L-shaped |
|  |  |  |  |  | Standardized differences* | -3.151 | **<0.001** | -0.76 | 0.224 | -1.084 | 0.139 |  |
|  |  |  |  |  | Wilcoxon test (one-tail for deficiency i.e. expansion) |  | **0.004** |  | 0.244 |  | 0.180 |  |
|  |  |  |  |  | Wilcoxon test (one-tail for excess i.e. bottleneck) |  | 0.998 |  | 0.773 |  | 0.835 |  |
|  |  |  |  |  | Wilcoxon test (two-tail) |  | **0.008** |  | 0.489 |  | 0.359 |  |
| Western Wales RBD Region | 95 | 0.54 | No | No | Sign Test (No. loci with heterozygosity excess) | 2 | **<0.001** | 8 | 0.425 | 5 | 0.040 | Normal L-shaped |
|  |  |  |  |  | Standardized differences* | -2.852 | **0.002** | -0.661 | 0.254 | -0.97 | 0.167 |  |
|  |  |  |  |  | Wilcoxon test (one-tail for deficiency i.e. expansion) |  | **0.001** |  | 0.262 |  | 0.126 |  |
|  |  |  |  |  | Wilcoxon test (one-tail for excess i.e. bottleneck) |  | 0.999 |  | 0.756 |  | 0.885 |  |
|  |  |  |  |  | Wilcoxon test (two-tail) |  | **0.003** |  | 0.524 |  | 0.252 |  |
| Central England Cluster | 132 | 0.73 | No | Yes | Sign Test (No. loci with heterozygosity excess) | 7 | 0.227 | 9 | 0.578 | 9 | 0.586 | Normal L-shaped |
|  |  |  |  |  | Standardized differences* | -2.395 | **0.008** | 0.843 | 0.200 | 0.337 | 0.368 |  |
|  |  |  |  |  | Wilcoxon test (one-tail for deficiency i.e. expansion) |  | 0.084 |  | 0.849 |  | 0.738 |  |
|  |  |  |  |  | Wilcoxon test (one-tail for excess i.e. bottleneck) |  | 0.924 |  | 0.165 |  | 0.281 |  |
|  |  |  |  |  | Wilcoxon test (two-tail) |  | 0.169 |  | 0.330 |  | 0.561 |  |
| Central England Cluster | 112 | 0.73 | No | No | Sign Test (No. loci with heterozygosity excess) | 7 | 0.235 | 10 | 0.370 | 8 | 0.413 | Normal L-shaped |
|  |  |  |  |  | Standardized differences* | -1.916 | **0.028** | 0.906 | 0.182 | 0.482 | 0.315 |  |
|  |  |  |  |  | Wilcoxon test (one-tail for deficiency i.e. expansion) |  | 0.076 |  | 0.916 |  | 0.820 |  |
|  |  |  |  |  | Wilcoxon test (one-tail for excess i.e. bottleneck) |  | 0.932 |  | 0.094 |  | 0.195 |  |
|  |  |  |  |  | Wilcoxon test (two-tail) |  | 0.151 |  | 0.188 |  | 0.389 |  |
| South West Cluster | 78 | 0.6 | No | Yes | Sign Test (No. loci with heterozygosity excess) | 3 | **0.002** | 7 | 0.062 | 7 | 0.232 | Normal L-shaped |
|  |  |  |  |  | Standardized differences* | -3.804 | **<0.001** | -1.096 | 0.136 | -1.52 | 0.064 |  |
|  |  |  |  |  | Wilcoxon test (one-tail for deficiency i.e. expansion) |  | **<0.001** |  | 0.262 |  | 0.151 |  |
|  |  |  |  |  | Wilcoxon test (one-tail for excess i.e. bottleneck) |  | 1.000 |  | 0.756 |  | 0.8616 |  |
|  |  |  |  |  | Wilcoxon test (two-tail) |  | **0.001** |  | 0.524 |  | 0.303 |  |
| South West Cluster | 65 | 0.59 | No | No | Sign Test (No. loci with heterozygosity excess) | 1 | **<0.001** | 8 | 0.423 | 7 | 0.238 | Normal L-shaped |
|  |  |  |  |  | Standardized differences* | -4.011 | **<0.001** | -1.369 | 0.086 | -1.684 | **0.046** |  |
|  |  |  |  |  | Wilcoxon test (one-tail for deficiency i.e. expansion) |  | **<0.001** |  | 0.244 |  | 0.151 |  |
|  |  |  |  |  | Wilcoxon test (one-tail for excess i.e. bottleneck) |  | 1.000 |  | 0.773 |  | 0.862 |  |
|  |  |  |  |  | Wilcoxon test (two-tail) |  | **<0.001** |  | 0.489 |  | 0.303 |  |
| Wales and Borders Cluster | 186 | 0.57 | No | Yes | Sign Test (No. loci with heterozygosity excess) | 1 | **<0.001** | 3 | **0.002** | 3 | **0.002** | Normal L-shaped |
|  |  |  |  |  | Standardized differences* | -9.155 | **<0.001** | -4.064 | **<0.001** | -4.769 | **<0.001** |  |
|  |  |  |  |  | Wilcoxon test (one-tail for deficiency i.e. expansion) |  | **<0.001** |  | **0.002** |  | **<0.001** |  |
|  |  |  |  |  | Wilcoxon test (one-tail for excess i.e. bottleneck) |  | 1.000 |  | 0.999 |  | 0.999 |  |
|  |  |  |  |  | Wilcoxon test (two-tail) |  | **<0.001** |  | **0.003** |  | **0.002** |  |
| Wales and Borders Cluster | 170 | 0.56 | No | No | Sign Test (No. loci with heterozygosity excess) | 1 | **<0.001** | 3 | **0.002** | 3 | **0.002** | Normal L-shaped |
|  |  |  |  |  | Standardized differences* | -7.325 | **<0.001** | -2.964 | **0.002** | -3.529 | **<0.001** |  |
|  |  |  |  |  | Wilcoxon test (one-tail for deficiency i.e. expansion) |  | **<0.001** |  | **0.011** |  | **0.005** |  |
|  |  |  |  |  | Wilcoxon test (one-tail for excess i.e. bottleneck) |  | 1.000 |  | 0.991 |  | 0.996 |  |
|  |  |  |  |  | Wilcoxon test (two-tail) |  | **<0.001** |  | **0.022** |  | **0.010** |  |
| *Fewer than the required 20 loci | |  |  |  |  |  |  |  |  |  |  |  |

### **Table S2: Temporally restricted Bottleneck results using data from 2009 and 2014 only.** N: number of individuals analysed; Mean H_e_: the mean heterozygosity across samples in the dataset; Test, refers to the specific test within Bottleneck; Test Stat, is the test statistic; p-value, is the p-value of the given test; SMM, single mutation model; TPM (88%, 30), Two-Phase Model of mutation with 88% stepwise mutations and variance of 30; TPM (88%, 12), Two-Phase Model of mutation with 88% stepwise mutations and variance of 12; Mode Shift, the distribution of allele frequencies. Significant p-values (p<0.05) are highlighted in bold.

| **Dataset** | **N** | **Mean H_e_** | **Test** | **SMM** | | **TPM (88%, 30)** | | **TPM (88%, 12)** | | **Mode Shift** |
| --- | --- | --- | --- | --- | --- | --- | --- | --- | --- | --- |
|  |  |  |  | **Test Stat** | **p- value** | **Test Stat** | **p- value** | **Test Stat** | **p- value** |  |
| Eastern | 43 | 0.71 | Sign Test (No. loci with heterozygosity excess) | 8 | 0.402 | 12 | 0.089 | 12 | 0.080 | Normal L-shaped |
|  |  |  | Standardized differences* | -0.876 | 0.191 | 0.999 | 0.159 | 0.798 | 0.212 |  |
|  |  |  | Wilcoxon test (one-tail for deficiency i.e. expansion) |  | 0.719 |  | 0.958 |  | 0.953 |  |
|  |  |  | Wilcoxon test (one-tail for excess i.e. bottleneck) |  | 0.300 |  | **0.047** |  | 0.054 |  |
|  |  |  | Wilcoxon test (two-tail) |  | 0.600 |  | 0.095 |  | 0.107 |  |
| Northern | 20 | 0.69 | Sign Test (No. loci with heterozygosity excess) | 8 | 0.411 | 9 | 0.596 | 9 | 0.603 | Normal L-shaped |
|  |  |  | Standardized differences* | -1.879 | 0.030 | -0.201 | 0.420 | -0.36 | 0.361 |  |
|  |  |  | Wilcoxon test (one-tail for deficiency i.e. expansion) |  | 0.195 |  | 0.533 |  | 0.511 |  |
|  |  |  | Wilcoxon test (one-tail for excess i.e. bottleneck) |  | 0.820 |  | 0.489 |  | 0.511 |  |
|  |  |  | Wilcoxon test (two-tail) |  | 0.389 |  | 0.978 |  | 1.000 |  |
| South West** | 37 | 0.58 | Sign Test (No. loci with heterozygosity excess) | 2 | **<0.001** | 4 | **0.013** | 4 | **0.013** | Normal L-shaped |
|  |  |  | Standardized differences* | -3.448 | **<0.001** | -1.415 | 0.079 | -1.77 | **0.040** |  |
|  |  |  | Wilcoxon test (one-tail for deficiency i.e. expansion) |  | **<0.001** |  | **0.024** |  | **0.013** |  |
|  |  |  | Wilcoxon test (one-tail for excess i.e. bottleneck) |  | 1.000 |  | 0.979 |  | 0.989 |  |
|  |  |  | Wilcoxon test (two-tail) |  | **<0.001** |  | **0.048** |  | **0.026** |  |
| Severn | 22 | 0.54 | Sign Test (No. loci with heterozygosity excess) | 5 | **0.041** | 7 | 0.234 | 6 | 0.121 | Normal L-shaped |
|  |  |  | Standardized differences* | -1.867 | **0.031** | -0.565 | 0.286 | -0.69 | 0.246 |  |
|  |  |  | Wilcoxon test (one-tail for deficiency i.e. expansion) |  | 0.054 |  | 0.340 |  | 0.262 |  |
|  |  |  | Wilcoxon test (one-tail for excess i.e. bottleneck) |  | 0.953 |  | 0.681 |  | 0.756 |  |
|  |  |  | Wilcoxon test (two-tail) |  | 0.107 |  | 0.679 |  | 0.524 |  |
| Western Wales | 31 | 0.56 | Sign Test (No. loci with heterozygosity excess) | 5 | 0.036 | 8 | 0.420 | 8 | 0.417 | Normal L-shaped |
|  |  |  | Standardized differences* | -1.13 | 0.129 | 0.211 | 0.416 | 0.042 | 0.483 |  |
|  |  |  | Wilcoxon test (one-tail for deficiency i.e. expansion) |  | 0.068 |  | 0.756 |  | 0.555 |  |
|  |  |  | Wilcoxon test (one-tail for excess i.e. bottleneck) |  | 0.940 |  | 0.262 |  | 0.467 |  |
|  |  |  | Wilcoxon test (two-tail) |  | 0.135 |  | 0.524 |  | 0.934 |  |
| *Fewer than the required 20 loci | | | | | | | | | | |

### **Table S3: Temporally restricted Bottleneck results for the Wales and Borders Region.** Years: the years included in the analysis; N: number of individuals analysed; Mean H_e_: the mean heterozygosity across samples; Test: the specific test within BOTTLENECK; Test Stat: the test statistic; p-value: the p-value of the given test; SMM: stepwise mutation model; TPM (88%, 30), Two-Phase mutation Model with 88% stepwise mutations and variance of 30; TPM (88%, 12): Two-Phase mutation Model with 88% stepwise mutations and variance of 12; Mode Shift: the BOTTLENECK indicator of the distribution of allele frequencies. Significant p-values (p<0.05) are highlighted in bold.

| **Dataset** | **Years** | **N** | **Mean H_e_** | **Test** | **SMM** | | **TPM (88%, 30)** | | **TPM (88%, 12)** | | **Mode Shift** |
| --- | --- | --- | --- | --- | --- | --- | --- | --- | --- | --- | --- |
|  |  |  |  |  | **Test Stat** | **p-value** | **Test Stat** | **p-value** | **Test Stat** | **p-value** |  |
| Wales and Borders | 1993-1995 | 25 | 0.50 | Sign Test (No. loci with heterozygosity excess) | 7 | 0.226 | 9 | 0.560 | 8 | 0.445 | Normal L shaped |
|  |  |  |  | Standardized differences* | -1.039 | 0.149 | 0.042 | 0.483 | -0.176 | 0.430 |  |
|  |  |  |  | Wilcoxon test (one-tail for deficiency i.e. expansion) |  | 0.262 |  | 0.661 |  | 0.577 |  |
|  |  |  |  | Wilcoxon test (one-tail for excess i.e. bottleneck) |  | 0.756 |  | 0.360 |  | 0.445 |  |
|  |  |  |  | Wilcoxon test (two-tail) |  | 0.524 |  | 0.720 |  | 0.890 |  |
| Wales and Borders | 2014 | 28 | 0.53 | Sign Test (No. loci with heterozygosity excess) | 3 | 0.002 | 6 | 0.112 | 5 | 0.039 | Normal L shaped |
|  |  |  |  | Standardized differences* | -3.005 | 0.001 | -1.202 | 0.115 | -1.462 | 0.072 |  |
|  |  |  |  | Wilcoxon test (one-tail for deficiency i.e. expansion) |  | 0.006 |  | 0.104 |  | 0.060 |  |
|  |  |  |  | Wilcoxon test (one-tail for excess i.e. bottleneck) |  | 0.995 |  | 0.906 |  | 0.947 |  |
|  |  |  |  | Wilcoxon test (two-tail) |  | 0.012 |  | 0.208 |  | 0.121 |  |
| Wales and Borders | 1993-1999 | 59 | 0.52 | Sign Test (No. loci with heterozygosity excess) | 6 | 0.100 | 8 | 0.424 | 8 | 0.431 | Normal L shaped |
|  |  |  |  | Standardized differences* | -1.819 | 0.034 | -0.299 | 0.382 | -0.387 | 0.350 |  |
|  |  |  |  | Wilcoxon test (one-tail for deficiency i.e. expansion) |  | 0.068 |  | 0.381 |  | 0.360 |  |
|  |  |  |  | Wilcoxon test (one-tail for excess i.e. bottleneck) |  | 0.940 |  | 0.640 |  | 0.661 |  |
|  |  |  |  | Wilcoxon test (two-tail) |  | 0.135 |  | 0.762 |  | 0.720 |  |
| Wales and Borders | 2009-2014 | 53 | 0.56 | Sign Test (No. loci with heterozygosity excess) | 2 | <0.001 | 4 | 0.013 | 4 | 0.011 | Normal L shaped |
|  |  |  |  | Standardized differences* | -3.597 | <0.001 | -1.415 | 0.079 | -1.648 | 0.050 |  |
|  |  |  |  | Wilcoxon test (one-tail for deficiency i.e. expansion) |  | <0.001 |  | **0.024** |  | **0.015** |  |
|  |  |  |  | Wilcoxon test (one-tail for excess i.e. bottleneck) |  | 1.000 |  | 0.980 |  | 0.987 |  |
|  |  |  |  | Wilcoxon test (two-tail) |  | <0.001 |  | **0.048** |  | **0.030** |  |
| *Fewer than the required 20 loci | | | | | | | | | | | |

### **Table S4: Spatially explicit estimates of effective population size (Ne) based on single-sample linkage disequilibrium (LD) methods with and without accounting for geographic population structure and admixed individuals.** Dataset: the geographic or genetic extent of the data; Admixed Individuals: included (Yes) or excluded (No) based on a q<0.8 assignment in STRUCTURE to one of the three genetic clusters; N: number of analysed individuals; N_e_: the effective population size estimate; L95% and U95%: the lower and upper 95% confidence interval, respectively, as determined through jackknifing; P_crit_: the critical value for exclusion of rare alleles (based on sample size using the formula: 1/(2 x N) < P_crit_ < 1/N).

| **Dataset** | **Admixed individuals** | **N** | **N_e_** | **L95%** | **U95%** | **P_crit_** |
| --- | --- | --- | --- | --- | --- | --- |
| All Data | Yes | 407 | 47.8 | 41.1 | 55.5 | None |
| Wales and England | Yes | 396 | 45.4 | 38.9 | 52.8 | None |
| Eastern England RBD | Yes | 74 | 41.7 | 29.7 | 61.7 | 0.01 |
| Northern England RBD | Yes | 59 | 81.8 | 50.6 | 168.9 | 0.01 |
| South West England RBD | Yes | 77 | 25 | 13 | 51.8 | 0.01 |
| Western Wales & Severn RBDs | Yes | 186 | 37.1 | 24.5 | 56.3 | None |
| Sum RBD estimates | Yes | 396 | 185.6 | 117.8 | 338.7 |  |
| Central England Cluster | Yes | 132 | 64.6 | 51.6 | 82.5 | None |
| South West England Cluster | Yes | 78 | 22.7 | 16.1 | 32.5 | 0.01 |
| Wales and Borders Cluster | Yes | 186 | 39.4 | 27.4 | 57.1 | None |
| Sum Cluster Estimates | Yes | 396 | 126.7 | 95.1 | 172.1 |  |
| Wales and England | No | 330 | 40.7 | 34.6 | 47.7 | None |
| Eastern England RBD | No | 64 | 41.5 | 28.2 | 66.2 | 0.01 |
| Northern England RBD | No | 42 | 56.1 | 31.4 | 147.6 | 0.02 |
| South West England RBD | No | 58 | 30.7 | 18.2 | 58.2 | 0.01 |
| Western Wales & Severn RBDs | No | 166 | 42.3 | 24.3 | 76.3 | None |
| Sum RBD estimates | No | 330 | 170.6 | 102.1 | 348.3 |  |
| Wales and England | No | 347 | 41.3 | 35.2 | 48.4 | None |
| Central England Cluster | No | 112 | 59.1 | 46.7 | 76.7 | None |
| South West England Cluster | No | 65 | 21.3 | 13.6 | 34.2 | 0.01 |
| Wales and Borders Cluster | No | 170 | 40.9 | 28.1 | 60.3 | None |
| Sum Cluster Estimates | No | 347 | 121.3 | 88.4 | 171.2 |  |

### **Table S5: Temporal estimates of effective population size (Ne) based on single-sample linkage disequilibrium (LD) methods with and without accounting for geographic population structure and admixed individuals.** Time group: temporal spread of the samples included in the analysis (Early ≤ 2004, late ≥ 2009); N: number of individuals analysed; N_e_: effective population size estimate; L95% and U95%: the lower and upper 95% confidence intervals as determined through jackknifing; P_crit_: the critical value for exclusion of rare alleles (based on sample size using the formula: 1/(2 x N) < P_crit_ < 1/N).

| **Dataset** | **Time group** | **N** | **N_e_** | **L95%** | **U95%** | **P_crit_** |
| --- | --- | --- | --- | --- | --- | --- |
| Wales and England | All | 330 | 40.7 | 34.6 | 47.7 | None |
| Eastern England RBD | All | 64 | 41.5 | 28.2 | 66.2 | 0.01 |
| Northern England RBD | All | 42 | 56.1 | 31.4 | 147.6 | 0.02 |
| South West England RBD | All | 58 | 30.7 | 18.2 | 58.2 | 0.01 |
| Western Wales & Severn RBDs | All | 166 | 42.3 | 24.3 | 76.3 | None |
| **Sum RBD estimates** | **All** | **330** | **170.6** | **102.1** | **348.3** |  |
| Wales and England | All | 347 | 41.3 | 35.2 | 48.4 | None |
| Central England Cluster | All | 112 | 59.1 | 46.7 | 76.7 | None |
| South West England Cluster | All | 65 | 21.3 | 13.6 | 34.2 | 0.01 |
| Wales and Borders Cluster | All | 170 | 40.9 | 28.1 | 60.3 | None |
| **Sum Cluster Estimates** | **All** | **347** | **121.3** | **88.4** | **171.2** |  |
| Wales and England | Early | 177 | 24.9 | 21.2 | 29.2 | None |
| Eastern England RBD | Early | 21 | 21.7 | 13.2 | 43.6 | 0.02 |
| Northern England RBD | Early | 22 | 42.3 | 24.4 | 111.5 | 0.02 |
| South West England RBD | Early | 21 | 13.5 | 6.7 | 33.3 | 0.02 |
| Western Wales & Severn RBDs | Early | 113 | 22.3 | 8.6 | 55.1 | None |
| **Sum RBD estimates** | **Early** | **177** | **99.8** | **52.9** | **243.5** |  |
| Wales and England | Early | 185 | 24.8 | 21.1 | 29 | None |
| Central England Cluster | Early | 45 | 39.2 | 28.2 | 58.4 | 0.02 |
| South West England Cluster | Early | 26 | 10.2 | 4.6 | 22.6 | 0.02 |
| Wales and Borders Cluster | Early | 114 | 21.2 | 8.8 | 48.2 | None |
| **Sum Cluster Estimates** | **Early** | **185** | **70.6** | **41.6** | **129.2** |  |
| Wales and England | Late | 153 | 41.8 | 34.1 | 51.6 | None |
| Eastern England RBD | Late | 43 | 53.9 | 32.2 | 117.7 | 0.02 |
| Northern England RBD | Late | 20 | 29.6 | 10.6 | Inf | 0.05 |
| South West England RBD | Late | 37 | 39.1 | 19.6 | 129.2 | 0.02 |
| Western Wales & Severn RBDs | Late | 53 | 52.1 | 18.2 | Inf | 0.01 |
| **Sum RBD estimates** | **Late** | **153** | **174.7** | **80.6** | **Inf** |  |
| Wales and England | Late | 162 | 43 | 35.2 | 52.7 | None |
| Central England Cluster | Late | 67 | 45.7 | 33 | 67.1 | 0.01 |
| South West England Cluster | Late | 39 | 30.2 | 15.7 | 78.5 | 0.02 |
| Wales and Borders Cluster | Late | 56 | 35.9 | 15 | 155.7 | 0.01 |
| **Sum Cluster Estimates** | **Late** | **162** | **111.8** | **63.7** | **301.3** |  |
